# Electrical Stimulation of Coleopteran Muscle for Initiating Flight

**DOI:** 10.1371/journal.pone.0151808

**Published:** 2016-04-06

**Authors:** Hao Yu Choo, Yao Li, Feng Cao, Hirotaka Sato

**Affiliations:** School of Mechanical and Aerospace Engineering, Nanyang Technological University, Singapore, Singapore; Duke University, UNITED STATES

## Abstract

Some researchers have long been interested in reconstructing natural insects into steerable robots or vehicles. However, until recently, these so-called cyborg insects, biobots, or living machines existed only in science fiction. Owing to recent advances in nano/micro manufacturing, data processing, and anatomical and physiological biology, we can now stimulate living insects to induce user-desired motor actions and behaviors. To improve the practicality and applicability of airborne cyborg insects, a reliable and controllable flight initiation protocol is required. This study demonstrates an electrical stimulation protocol that initiates flight in a beetle (*Mecynorrhina torquata*, Coleoptera). A reliable stimulation protocol was determined by analyzing a pair of dorsal longitudinal muscles (DLMs), flight muscles that oscillate the wings. DLM stimulation has achieved with a high success rate (> 90%), rapid response time (< 1.0 s), and small variation (< 0.33 s; indicating little habituation). Notably, the stimulation of DLMs caused no crucial damage to the free flight ability. In contrast, stimulation of optic lobes, which was earlier demonstrated as a successful flight initiation protocol, destabilized the beetle in flight. Thus, DLM stimulation is a promising secure protocol for inducing flight in cyborg insects or biobots.

## Introduction

The development of reliable micro air vehicles (MAVs) has challenged researchers for decades and remains actively studied today. MAVs fly and navigate into restricted and complicated spaces with flexibility and splendid controllability. Therefore, MAVs that are practically usable in real life, especially in search-and-rescue operations and indoor surveillance [1], have been a long-term ambition of researchers. As micro system technologies advance, achieving this ambition has become increasingly realistic [2–4], and researchers have developed MAVs that are smaller and more controllable. However, even state-of-the-art MAVs cannot be used over long durations with complex maneuverability because of the limited energy capacity of the power source, high power consumption rate, and complicate control systems adopted for maintaining and stabilizing the posture in air [3].

Meanwhile, insect flight mechanisms and their aerodynamic characteristics have attracted considerable interest [5–7]. The efficient motors (flight muscles) of insects enable wing flapping over a long duration and subtle alterations in the wing beat trajectory, ensuring high maneuverability in air [8]. This raises the following question: could a live insect be adopted as an MAV platform; that is, could we mount or implant a tiny electrical stimulator on a live insect, thus controlling its motor actions by stimulating its neuromuscular sites? Such insect–machine hybrids, or biobots, have been actively researched [9–23]. Various methods have proved effective for controlling different types of insects, such as electrical [9, 11–13, 16, 19, 20, 23], photic [17], and thermal stimulation [10] as well as chemical injection [15]. By combining artificial devices with live insects, we can exploit the intrinsic excellent flight performance of insects to serve human needs. For example, insect–machine hybrid air vehicles can potentially monitor narrow and hazardous environments that are inaccessible to humans.

The first and most essential challenge of developing an insect–machine hybrid air vehicle is establishing a stable flight initiation protocol for the insects. A flight initiation protocol is a requisite of a fully controlled air vehicle. Such a protocol should be highly reliable, rapidly responsive, and minimally destructive. Several methods of flight initiation have been proposed for various insects, each with its advantages and disadvantages. Specifically, cockroach flight has been chemically stimulated by octopamine and wind puff [15]. Moth flight has been successfully initiated by electrical stimulation of the brain and thorax [9]. Other researchers have electrically stimulated the optic lobes of beetle heads to initiate flight [11, 12]. Beetle flight has also been accomplished by micro-thermal stimulation at the base of the antenna [10]. Among these methods, electrical stimulation appears to be the most suitable in practice, because it is easily applied and delivers highly reliable results. However, as electrical stimulations to the head area require accurate microsurgery skills and may permanently damage the insect body, electrical stimulations at parts other than the head, for example, the thorax, should be the next focus of flight initiation. Electrodes cannot be precisely implanted and fixed in neuronal tissue, because the tiny, densely arrayed neurons are difficult to separate. In contrast, muscles are much larger and easily identified under a conventional optical microscope or even by the naked eye. Thus we have selected muscle as the target of electrical stimulation to induce our desired motor action, flight initiation. The primary outcome from this study is negligible damage flight initiation with high success rate > 90%. The beetle species *Mecynorrhina torquata* has a relatively large body size and a high load capacity (in flight, it can carry 20–30% of its body weight) [11, 24]. Thus, this species is a suitable platform for cyborg insect or biobot. We investigated the indirect flight muscles, namely, the dorsal longitudinal muscles (DLMs) and dorso ventral muscles (DVMs), which generate the wing oscillation [5, 25]. To initiate flight, we attempted to stimulate either of these muscles with electrical pulses.

## Materials and Methods

### Study Insect

All experiments were performed on specimens of *M*. *torquata* (order Coleoptera; length: 62 ± 8 mm; mass: 7.7 ± 1.9 g). The beetles were kept in separate plastic terrariums with wood bedding and fed with beetle jelly twice a week. Specimens with no obvious visible defects were chosen for the flight initiation experiments. The experimental room was maintained at 29°C, a suitable temperature for beetle flight [26]. The use of this animal is permitted by the Agri-Food and Veterinary Authority of Singapore (AVA, HS code: 01069000, Product code: ALV002). Invertebrates, including insects, are exempt from ethics approval for animal experimentation according to the National Advisory Committee for Laboratory Animal Research (NACLAR) guidelines.

In order to judge whether given beetles could fly normally under a criterion that intact beetles can fly longer than 10 s [20], the natural flight ability of every beetle was tested in a free flight prior to all the experiments. Every beetle was thrown into the air to naturally initiate flight, and we checked whether the flight time was longer than 10 s. This free flight ability test was commonly conducted throughout this study to judge whether beetles can normally fly after we operated certain experiments such as removal of elytra or scutellum, blind folding, electrode implantation, and electrical stimulation of DLM or optic lobes (see the sections of Electrical Stimulation, Results and Discussion).

### Electrode Implantation

**Fig 1:**
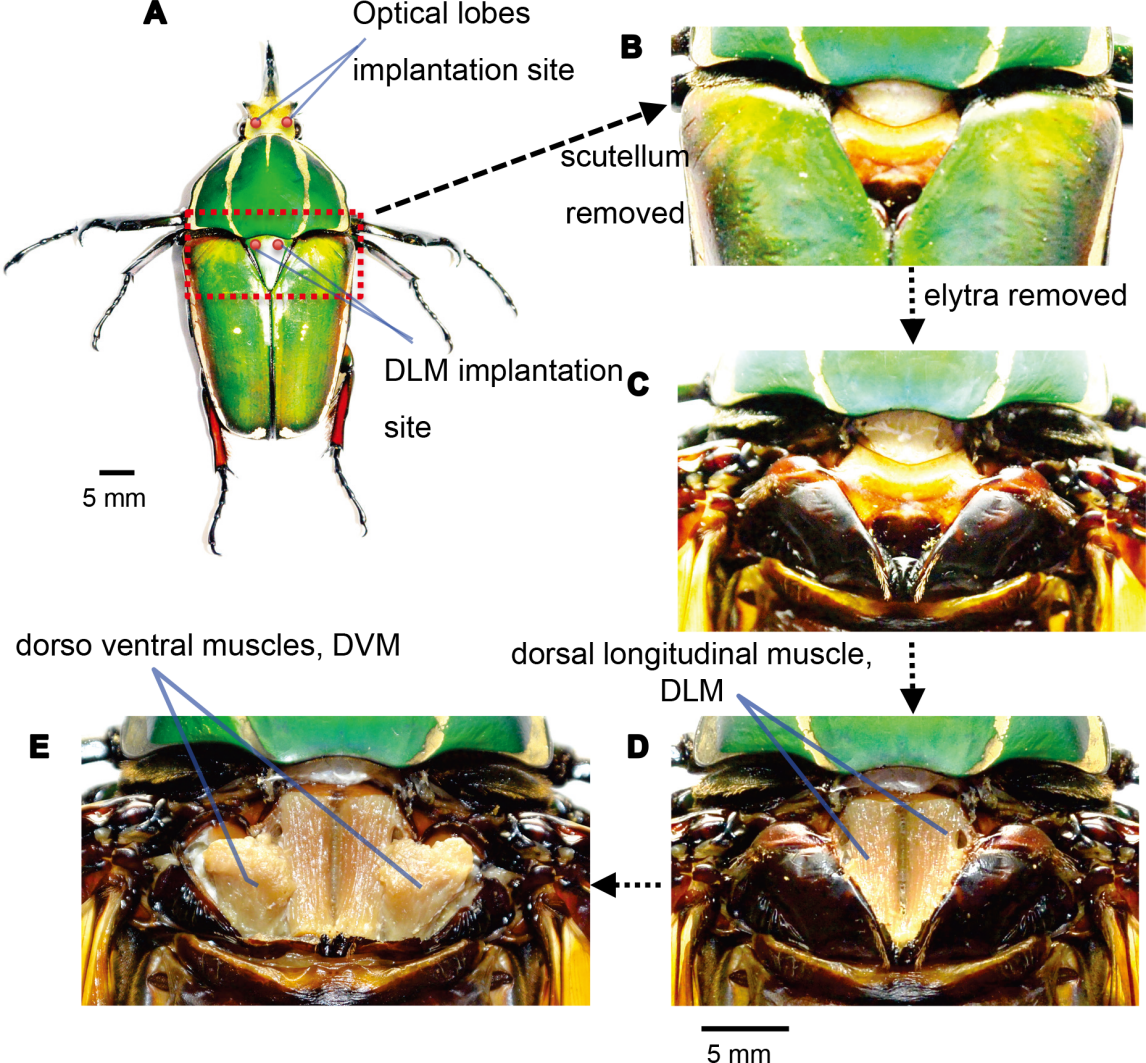
Anatomical view of pairs of the antagonistic flight muscles, namely, the dorsal longitudinal muscle (DLM) and dorsal ventral muscle (DVM). (A) Overview of the dorsal side of a beetle, with the locations of implantation site at scutellum and head. The electrodes go through the holes made in the scutellum into DLM and head into optical lobes. Magnified views of dorsal thorax after (B) removal of scutellum, (C) removal of elytra, (D) exposing DLM, and (E) exposing DVM.

Thin Teflon-insulated silver wires (A-M Systems; uncoated diameter, 127 μm; Teflon-coated diameter; 178 μm) were used as electrode for stimulation. Both ends of a silver wire were burned in a flame to remove the Teflon insulation layer. For the stimulation of DLM, a pair of silver wires was 4 mm inserted into the holes which were pierced beneath the scutellum (Fig 1A). For the stimulation of optical lobes, a pair of silver wires was 2 mm inserted into the holes pierced near the left and right compound eyes as shown in Fig 1A according to [11]. The implanted electrodes were then glued with beeswax. The other ends of the implanted wires were connected to the output signal port and to the ground (GND) port of a function generator (Agilent, 33220A).

### Electrical Stimulation

Every tested beetle was tethered using a 20-cm-long stick, which was vertically clamped to a plane table by a magnetic base. The lower tip of the stick mounted a small cubical magnet while another magnet was glued on the pronotum of the beetle. The beetle was held under the stick with these magnets. The tethered beetle could move neither horizontally nor vertically. Pulse train stimulation signal with 2.0 or 3.0 V in amplitude (2.0 V for optic lobe, 3.0 V for DLM), 100 Hz in frequency and 10% in duty cycle were applied to optic lobe or DLM by the function generator. The stimulation signals were monitored by an oscilloscope (Yokogawa, DL1640).

The stimulated beetle was monitored for 5 s after the stimulation signal was output to the beetle, which was filmed at 30 frames per second. If the stimulated beetle unfolds and oscillates the wings within the 5 s, it is counted as a success in the flight initiation. For every tested beetle, this stimulation and monitoring were repeated 10 times. To avoid exhaustion of the tested beetles and to judge fairly on the success/failure of the flight initiation at every trial, even if the electrical stimulation successfully initiated flight, we stopped the flight by softly touching the wings.

**Fig 2:**
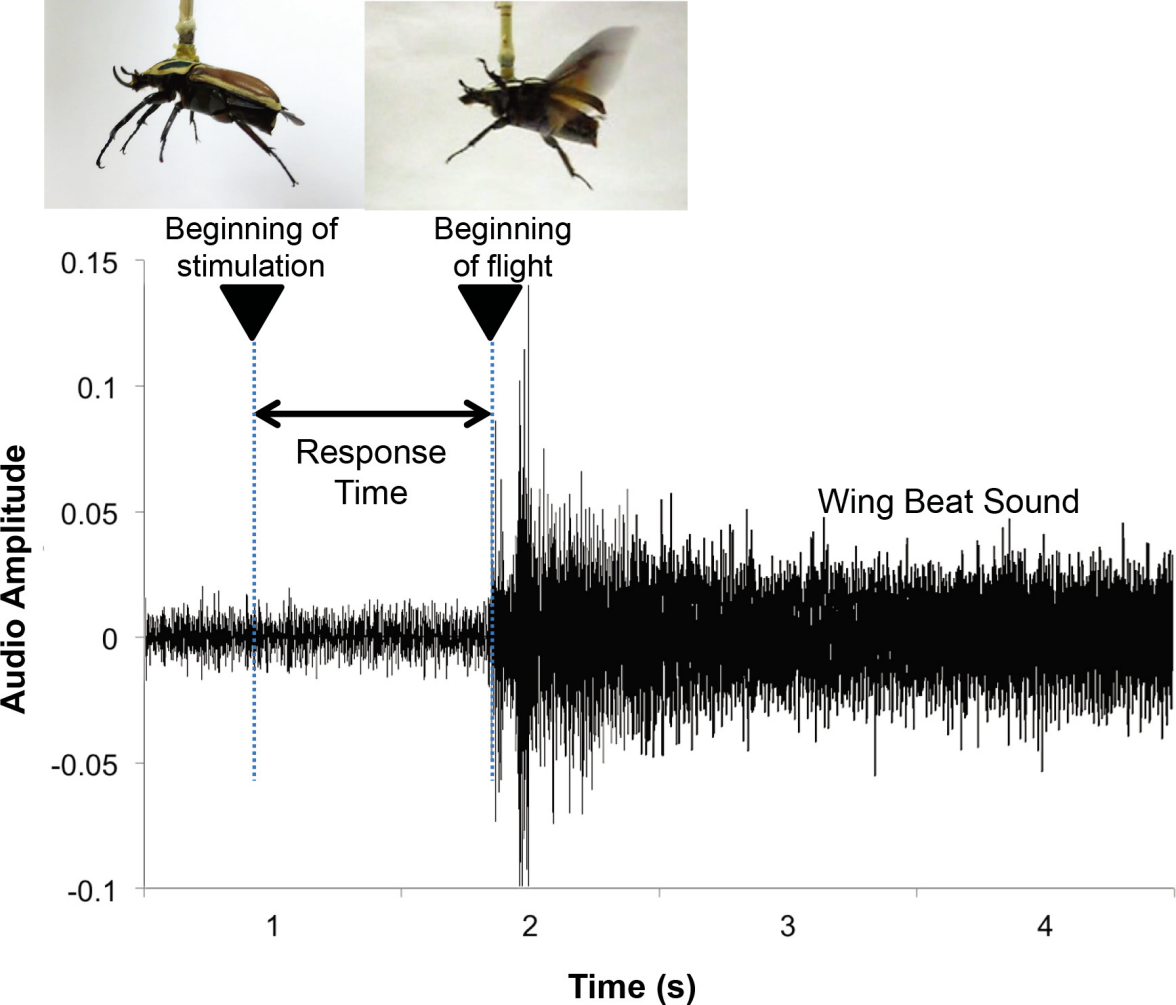
The response time is the elapsed time from the beginning of the electrical stimulation to the beginning of flight (first wing beat). The response time was counted by means of frame-by-frame playback between the trigger and the first wing beat, as recorded by a video recorder.

The rate of the number of success in the flight initiation to the number of trials is defined as the success rate. The response time was determined by means of frame-by-frame playback to count the number of frames between the stimulation signal trigger (beginning of stimulation) and the first wing beat (beginning of flight) as seen in Fig 2. The timing of the trigger is determined by the display of the oscilloscope or the sound marker from the function generator. The sound marker did not affect the flight initiation. No beetle reacted to the sound marker to unfold the wings (N = 5 animals, n = 100 trials). The stimulation was followed by the damage extent test (free flight ability test) to judge whether the electrical stimulation led to crucial damage to the beetle flight ability. After the stimulation experiment, each beetle was thrown into the air to naturally initiate flight. If the beetle can fly for longer than 10 s, it is counted as a pass in the damage extent test. Otherwise it is counted as a failure.

## Results and Discussion

As the optic lobes constitute the massive neural cluster of the compound eye, the electrical stimulation would likely destabilize the beetle’s flight. Following Sato et al. [11], we implanted the stimulation electrodes into the left and right optic lobes and applied electrical stimulation (2 V, 100 Hz, N = 5 animals, n = 50 trials). As reported in [11], the beetles unfolded and began oscillating their wings due to the stimulation. In the free flight ability test (see the section Study Insect), all the stimulated beetles lost steerage in the air and could not sustain flight for 10 s (N = 5 animals, n = 25 trials). Beetles thrown into the air usually fly spontaneously (typically, they unfold their wings, begin wing oscillation, and fly for more than 10 s).

The same reaction (unfolding, oscillating but losing flight control) was confirmed in beetles that were blindfolded by sealing their compound eyes with beeswax and plasticine (N = 5 animals, n = 25 trials). When the blindfold was removed, all the beetles recovered the flight ability and flew more than 10 s in the free flight ability test. We conclude that the electrical stimulation causes flight disturbance by crucially damaging the optic lobe. As optical lobes are upstream of the neural network system in animals [27], their damage will disrupt muscles downstream of the neural network terminal. Thus, we questioned whether stimulating the flight muscles rather than the optic lobes would initiate flight without incurring crucial damage.

Beetles and many other insect orders maintain wing oscillations by alternately contracting their DVM and DLM, which constitute an antagonistic pair of flight muscles. To initiate wing oscillations, either or both the DVM and DLM should be stimulated. The DVM and DLM are located in a side domain and mid-domain, respectively, in the thorax of a beetle (Fig 1E). To implant something into DVM, the elytra needs to be cut and removed to expose the cuticle enclosing the DVM (Fig 1). We note that the elytra play a critical role in flight steerage. The elytra of other coleopteran generate lift during flight [28, 29] and the elytra form part of the mechanism that folds the hind wings [30, 31].

In fact, the removal of the elytra resulted in loss of steerage. Two days following the removal of their elytra, 4 out of 5 beetles lost their flight ability within 10 s; that is, 80% of the tested beetles demonstrated significantly impaired flight ability. We also note that, since the DVM is inserted in the cuticle (Fig 1D), part of that cuticle is destroyed by the electrode implantation, reducing the power output of the DVM. Eventually, we concluded the DVM is not an appropriate target for the electrical stimulation.

Another option for flight initiation is stimulation of DLM, the counterpart of the DLM–DVM antagonistic pair for wing oscillation. The DLM is located underneath the thin cuticle (Fig 1B), which is found underneath the thick, triangular-shaped cuticle referred as to scutellum (Fig 1A). Notably, unlike beetles with elytra removed, all the beetles with scutellum removed flew stably for more than 10 s even two days after the removal (N = 5 animals). The removal of the scutellum does not significantly affect the free flight ability. In addition, the DLM fibers are oriented parallel to the plane of the thin cuticle and the scutellum (the DLM is inserted into the internal cuticle plate perpendicular to the thin cuticle and the scutellum). Thus, implantation of the electrodes into the thin cuticle would not significantly reduce the power output of the DLM and would not result in the loss of flight ability. In fact, when electrodes were implanted into the DLM through holes pierced in the thin cuticle, the tested beetles exhibited no obvious irregular behavior during flight. All the beetles with electrode implanted into the DLM passed the free flight ability test (N = 5 animals, n = 25 trials).

The DLM-stimulated beetles unfolded and oscillated their wings (N = 9 animals, n = 90 trials). DLM stimulation initiated flight in 82 of the 90 trials, thus with an average success rate of 91%. Notably, in contrast to the optic lobe stimulation, none of the tested beetles lost steerage in air, confirming that DLM stimulation imparted no crucial damage to the muscles. We observed all the tested beetles behaved and survived as regularly and normally as intact beetles. All the tested beetles survived for more than 7 days.

The significance of sample size was determined through binomial test and t test. The binomial test with the significant level of 0.05 and expected successful rate of 85% is used to test the significance of the successful rate. If the proportion of the effective stimulation is greater than or equal to 85% and the p-value less than 0.05 (p < 0.05), the stimulation protocol is statistically verified as significant success. The effective stimulation was defined to have response time less than 1.5 seconds, which was checked by the t-test with the confidential interval of 95%. The binomial test indicated that the stimulation has the successful rate of 94% which is higher than the expected 85% (p = 0.005, N = 9 beetles, n = 90 trials). Moreover, the t-test also showed that the effective stimulation has the response time of 0.77 ± 0.39 seconds that is less than 1.5 seconds (p < 0.0001, N = 9 beetles, n = 90 trials). Overall, the stimulation protocol for the flight initiation demonstrated in this study was verified to be significantly successful under the abovementioned criteria.

**Fig 3:**
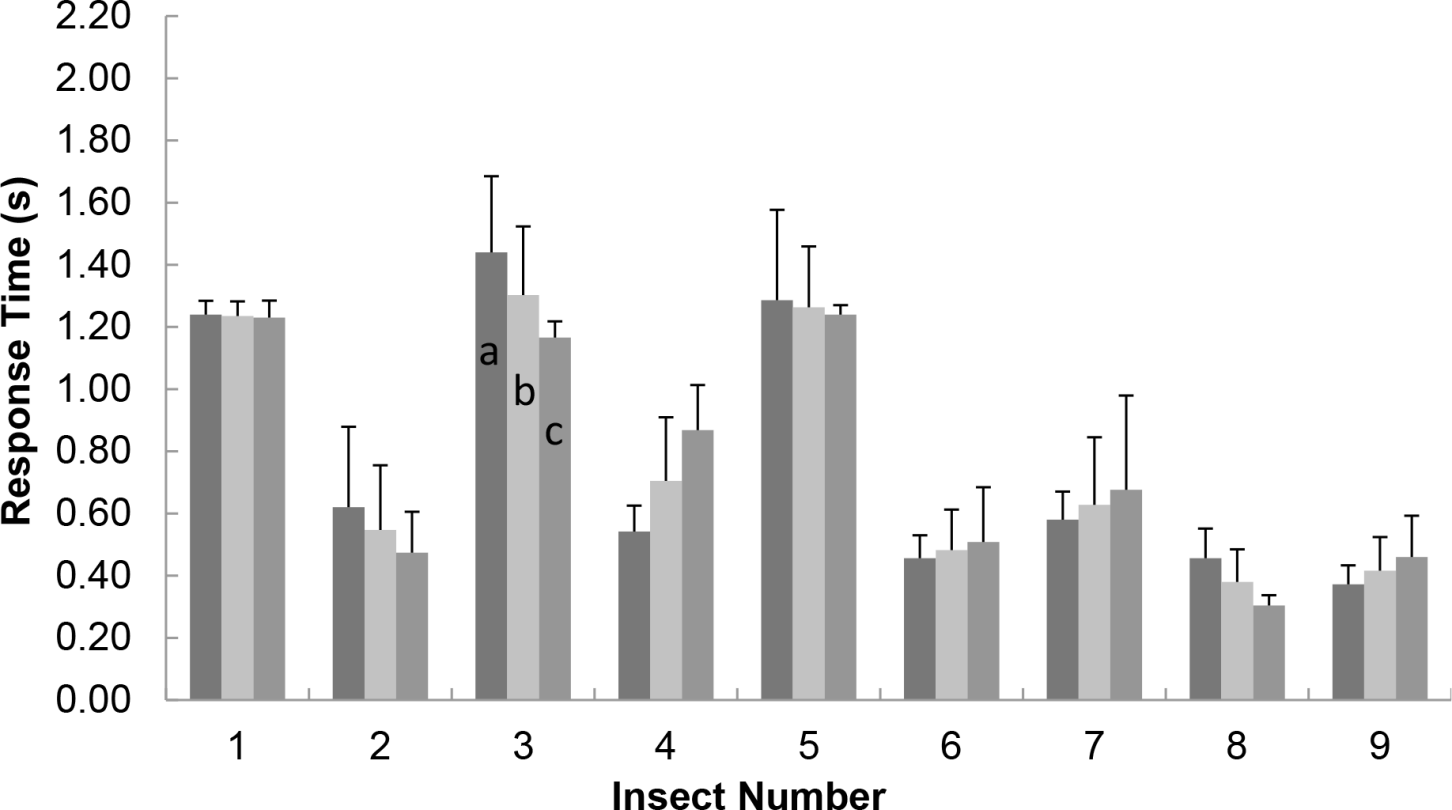
Response time of flight initiation. For each tested beetle, the (a) left, (b) middle, and (c) right columns indicate the average response times of the first 5 trials, all 10 trials, and the last 5 trials, respectively. The bar in each graph indicates the standard deviation.

The DLM stimulated beetles showed little habituation. We measured the response time to the DLM electrical stimulation, defined as the time interval between the beginning of the stimulation and the timing of wing unfolding (the beginning of wing oscillation) within a day, as illustrated in Fig 2. If it is measured over days and weeks, other factors such as electrode durability, animal vitality, electrode drift and feeding condition could affect the response time. In order to avoid such factors, the habituation test was conducted within a day. Significant habituation would manifest as lengthening response time; that is, the response time would increase as the stimulation was repeated. The average response times to the DLM stimulation in the first and second 5 trials differed by less than 0.33 s (Fig 3). Among all tested beetles and all trials, the response time varied by less than 23%. The beetles did not significantly become habituated to the DLM stimulation. The average response time was below 1.0 s, sufficiently short for practical application; specifically, this insect–machine hybrid is suitable for use as a miniature robot.

## Conclusion

According to the experimental results, beetle flight was initiated by applying 3 V electrical pulse signals at 100 Hz and 10% duty to the beetle DLM for 1 s. The success rate was considerably high (> 90%) and the damage due to the electrical stimulation is negligible for free flight ability. In conclusion, we successfully initiated a beetle’s wing beat by simple stimulation steps. Finally, we note that the wing-beat principles and muscle configurations of many insects are quite similar (namely, the down- and up-stroke of the wing is driven by the DLM and DVM, respectively) [5, 25]. Our approach might significantly contribute to the future design of insect–machine hybrid air vehicles.

Lastly, even though it might be a bit too early to mention potential medical applications, we wish the technologies and knowledge for insect-machine hybrid, for example, electrical stimulation of living muscles, to help some biomedical treatment, therapy and system such as functional electrical stimulation (FES), a technique to electrically activate nerves innervating extremities affected by paralysis to induce desired motor actions and behaviors of the patient.
